# Identification of potential biomarkers for lung cancer using integrated bioinformatics and machine learning approaches

**DOI:** 10.1371/journal.pone.0317296

**Published:** 2025-02-27

**Authors:** Md Symun Rabby, Md Merajul Islam, Sujit Kumar, Md Maniruzzaman, Md Al Mehedi Hasan, Yoichi Tomioka, Jungpil Shin

**Affiliations:** 1 Department of Statistics, Jatiya Kabi Kazi Nazrul Islam University, Trishal, Mymensingh, Bangladesh; 2 Statistics Discipline, Khulna University, Khulna, Bangladesh; 3 Department of Computer Science & Engineering, Rajshahi University of Engineering & Technology, Rajshahi, Bangladesh; 4 School of Computer Science and Engineering, The University of Aizu, Aizuwakamatsu, Japan; The First Hospital of Jilin University, CHINA

## Abstract

Lung cancer is one of the most common cancer and the leading cause of cancer-related death worldwide. Early detection of lung cancer can help reduce the death rate; therefore, the identification of potential biomarkers is crucial. Thus, this study aimed to identify potential biomarkers for lung cancer by integrating bioinformatics analysis and machine learning (ML)-based approaches. Data were normalized using the robust multiarray average method and batch effect were corrected using the ComBat method. Differentially expressed genes were identified by the LIMMA approach and carcinoma-associated genes were selected using Enrichr, based on the DisGeNET database. Protein-protein interaction (PPI) network analysis was performed using STRING, and the PPI network was visualized using Cytoscape. The core hub genes were identified by overlapping genes obtained from degree, betweenness, closeness, and MNC. Moreover, the MCODE plugin for Cytoscape was used to perform module analysis, and optimal modules were selected based on MCODE scores along with their associated genes. Subsequently, Boruta-based ML approach was utilized to identify the important genes. Consequently, the core genes were identified by the overlapping genes obtained from PPI networks, module analysis, and ML-based approach. The prognostic and discriminative power analysis of the core genes was assessed through survival and ROC analysis. We extracted five datasets from USA cohort and three datasets from Taiwan cohort and performed same experimental protocols to determine potential biomarkers. Four genes (*LPL, CLDN18, EDNRB, MME*) were identified from USA cohort, while three genes (*DNRB, MME, ROBO4*) were from Taiwan cohort. Finally, two biomarkers (*EDNRB* and *MME*) were identified by intersecting genes, obtained from USA and Taiwan cohorts. The proposed biomarkers can significantly improve patient outcomes by enabling earlier detection, precise diagnosis, and tailored treatment, ultimately contributing to better survival rates and quality of life for patients.

## Introduction

Lung cancer is one of the most common cancer and its prevalence and mortality rate have been rapidly increased globally. It is the leading cause of cancer-related death in both sexes [1]. Around 2.2 million new cases of lung cancer are diagnosed each year, and approximately 1.8 million people die from the disease worldwide [2]. There are two main subtypes of lung cancer: small-cell lung cancer (SCLC) and non-small cell lung cancer (NSCLC). NSCLC accounts for around 85% of patients, which is also the most malignant carcinoma among men and women [3–5]. It has grown to be a major worldwide health concern that has imposed a heavy financial burden on people and families. It is typically undiagnosed up to the advanced stages. While the survival rate for patients with lung cancer is quite low overall, there is a good possibility that they will get well if they receive appropriate diagnosis and treatment at an earlier stage. Treatment options for lung cancer depend on the type and stage of the cancer [6–8]. Despite considerable progress in lung cancer treatment, the mortality and recurrence rate in NSCLC patients are still not effectively controlled [9]. An accurate diagnosis and improved treatment have become increasingly required for the management of NSCLC patients in recent years. Therefore, identifying potential molecular biomarkers of NSCLC is essential for the early diagnosis and effective prognosis. Early diagnosis and effective/advanced treatment strategies can significantly enhance patient outcomes, thereby increasing the likelihood of survival and the quality of life for individuals with NSCLC.

Previously several studies have been carried out to identify the hub/core genes of NSCLC [9–19]. Most of them used only traditional bioinformatics approaches (for example PPI) to identify the hub genes. It is challenging to identify the hub genes for NSCLC at the genome level using conventional methods, which can occasionally yield misleading results, due to the complex pathways involving numerous crucial genes in the process. To address this issue, machine learning (ML) has recently gained significant popularity and attention across various fields, including bioinformatics [20–28]. The application of ML in bioinformatics allows for the analysis of large, complex genomic datasets, which are often high-dimensional and feature non-linear relationships. Unlike conventional methods, ML models are capable of processing vast amounts of data efficiently, uncovering hidden patterns, and identifying the most relevant genes associated with diseases like cancer. One of the key advantages of ML is its ability to develop effective prediction models. These models do not rely on predefined assumptions but rather learn directly from the data, making them more adaptable and capable of identifying novel relationships that may not be evident through traditional approaches. As a result, these models can determine more discriminative genes that have a stronger association with the disease, improving the precision and reliability of findings related to biomarkers for early detection, prognosis, and personalized treatment in diseases like NSCLC. Therefore, we proposed a potential biomarkers identification system for NSCLC by integrating bioinformatics analysis and ML-based approaches. The integration of this system is indeed powerful: bioinformatics tools allow for in-depth analysis of large-scale genomic data, while ML-based techniques excel at detecting complex patterns and relationships that may not be evident through conventional analysis. Moreover, this integrated approach enhances the robustness of our findings and offers a more comprehensive understanding of the underlying genetic factors associated with NSCLC. This advancement could lead to more precise diagnostic methods and facilitate the development of personalized therapeutic strategies. Therefore, the identified biomarkers not only provide valuable insights into the molecular mechanisms underlying the disease but also pave the way for the development of more accurate diagnostic tools and personalized therapesutic strategies, ultismately improving patient outcomes and guiding treatment decisions in clinical practice.

## Materials and methods

### Proposed methodology

**Fig 1:**
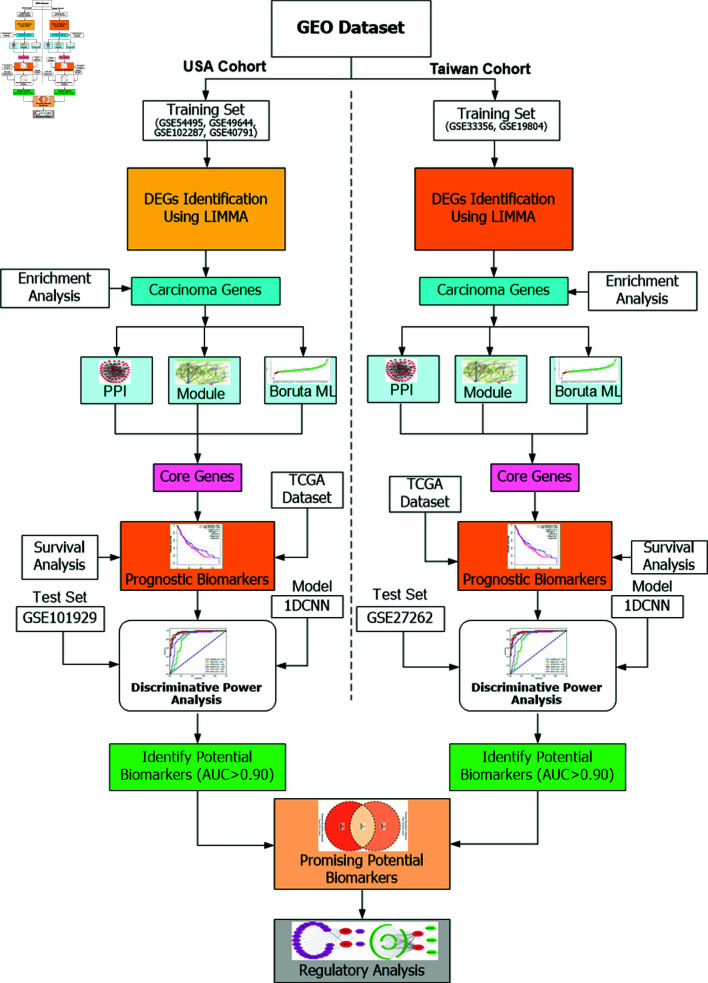
Overall working flowchart of promising potential biomarkers for NSCLC between USA cohort and Taiwan cohort. GEO: Gene expression omnibus; DEGs: Differentially expressed genes; LIMMA: Lnear models for microarray data; PPI: Protein-protein interaction; ML: Machine learning; TCGA: The cancer genome atlas; 1DCNN: One-dimensional convolutional neural network; AUC: Area under the curve.

The overall workflow adopted for this study is presented in Fig 1. In our study, we utilized gene expression omnibus (GEO) dataset derive from the USA and Taiwan cohort. The training dataset was employed to determine the core genes for each cohort of NSCLC and their performance was validated using test set. Firstly, we combined training datasets for each cohort and normalized them using robust multi-array average (RMA), followed by correction batch effect with the combat method. After that, we determined the differentually expressed genes (DEGs) by linear models for microarray data (LIMMA) and identified carcinema asssociated DEGs using Enrichr web tools for each cohrt. Subsequently, we applied the Database for annotation, visualization and integrated discovery (DAVID) for enrichment analysis that includes gene ontology (GO) and kyoto encyclopedia of genes and genomes (KEGG) pathway analysis. Following that, STRING was employed to perform the protein-protein interaction (PPI) network analysis and Cytoscape was used to determine the hub genes and cluster analysis. We determined the more important genes by Boruta based ML algorithm. The core genes then were identified by overlapping the genes, obtained from PPI networks, module analysis, and ML-based approach for each cohort. Subsequently, survival analysis of the core genes for each cohort was performed using data from The Cancer Genome Atlas (TCGA) through GEPIA and determined the prognostic biomarkers using p-value (<0.05). Moreover, discriminative power of the prognostic genes was evaluated using receiver operating characteristic (ROC) analysis by employing convolutional neural networks (CNN)-based model for each cohort. We subsequently identified the most promising potential biomarkers by intersecting genes, obtained from USA and Taiwan cohorts.

### Data acquisitions and preprocessing

This study used five microarray GEO data with accession numbers: GSE54495 [29], GSE49644 [30], GSE102287 [30], GSE40791 [31], and GSE101929 [32] https://www.ncbi.nlm.nih.gov/geo. The datasets were taken from the USA cohort with platform number GPL570. Four datasets (GSE54495, GSE49644, GSE102287, and GSE40791) were utilized as training set to identify the core genes of NSCLC, while another dataset (GSE101929) was used as a test set to validate their discriminative performance. Moreover, another three datasets with accession numbers: GSE33356 [33–35], GSE19804 [35,36], and GSE27262 [37,38]) based on GPL 570 (Affymetrix) platform were extracted from Taiwan cohort. Table 1 represents a detailed description of the datasets for USA and Taiwan cohort. The datasets were normalized using RMA normalization, which corrects for background noise and normalizes across arrays to stabilize expression levels. After normalization, the training datasets were combined, and batch effect were removed using the combat method [39]. The combat method effectively minimizes technical variation from different experimental conditions, ensuring that the combined datasets reflect true biological differences.

**Table 1:** Description of the datasets for USA and Taiwan cohort.

Datasets	Platform No.	Name	Country	Sample	Sex	NSCLC	Ctrl	Usage
GSE54495 [29]	GPL570	Affy.	USA	Lung	M/F	17	13	Training
GSE49644 [30]	GPL570	Affy.	USA	Lung	M/F	9	9	
GSE102287 [30]	GPL570	Affy.	USA	Lung	M/F	92	89	
GSE40791 [31]	GPL570	Affy.	USA	Lung	M/F	94	100	
GSE101929 [32]	GPL570	Affy.	USA	Lung	M/F	34	32	Test
GSE33356 [33–35]	GPL570	Affy.	Taiwan	Lung	F	60	60	
GSE19804 [35,36]	GPL570	Affy.	Taiwan	Lung	F	60	60	Training
GSE27262 [37,38]	GPL570	Affy.	Taiwan	Lung	NA	25	35	Test

Affy.: Affymetrix; M: Male; F: Female; NSCLC: Non-small cell lung cancer; Ctrl: Control.

### Identification of DEGs

The DEGs were identified for NSCLC using the LIMMA-based approach. LIMMA is a widely used method in bioinformatics for detecting DEGs, particularly well-suited to analyze high-throughput microarray data [40]. It applied linear modeling to detect genes with significant expression differences between NSCLC and normal tissues. This method enhances the detection power and accuracy of DEGs, highlighting genes potentially linked to NSCLC. In R programming, LIMMA was utilized. The DEGs were identified from the combined training dataset based on the following criteria: adjusted (Adj.) p-value  <  0.01 and  | $log_{2}$ fold change (FC) | > 2. The following cut-off points were adopted to separate the upregulated and downregulated DEGs:


$$\begin{array}{c} DEGs=\{\begin{array}{cc} Upregulated, & IfAdj.p-value<0.01\&\;\;{\text{Log}}_{2}FC>2\\ Downregulated, & IfAdj.p-value<0.01\&\;\;{\text{Log}}_{2}FC<-2 \end{array} \end{array}$$ (1)

### Disease genes association analysis

The carcinoma-associated gene in NSCLC among the DEGs were examined using the DisGeNET database through the Enrichr web-based tool (https://maayanlab.cloud/Enrichr/) [41,42]. DisGeNET is a comprehensive platform for gene discovery that includes information on genes and their variations associated with specific diseases. This study identified NSCLC gene associated with carcinoma using a criterion of Adj. p-value  <  0.05.

### Enrichment analysis

Enrichment analysis was performed to better understand the molecular mechanism and progression of carcinoma-associated genes in NSCLC. This analysis included three GO terms: biological process (BP), molecular function (MF), and cellular component (CC), along with KEGG pathways [43,44]. We utilized DAVID online tool (https://david.ncifcrf.gov/) to analyze significant GO terms and KEGG pathways related to NSCLC. The top significant GO-terms and KEGGG pathways were examined using a criterion of the Adj. p-value  <  0.05.

### PPI analysis and hub genes selection

PPI analysis was conducted to show the significant connection between the carcinoma associated genes by STRING (https://string-db.org) [45]. The confidence score  > 0.70 was used to make PPI among genes and constructed PPI network using the CytoHubba plugin in Cytoscape [46]. CytoHubba provides various topological measures, including degree (Deg), betweenness (Betn), closeness (Clos), and maximum neighborhood component (MNC) from the PPI networks [47]. The top 30 genes were then selected based on the value of each measures. Finally, the hub genes were determined by intersecting the genes, obtained from Deg, Betn, Clos, and MNC, respectively.

### Module analysis

Module analysis was employed to determine the prominent modules from the PPI network [48]. We adopted molecular complex detection (MCODE) in Cytoscape to perform module analysis with specific criteria: degree = 2, cluster finding = Haircut, nodes score cutoff = 0.30, K-score = 2, and max. depth = 100. The optimal modules were determined based on the value of MCODE scores  ( > 5 ) . Subsequently, we identified their correspondence nodes or genes from the determined modules.

### ML-based important genes identification

Boruta is a wrapper-based machine learning approach that makes use of the random forest (RF) classifier to identify the important genes [49–52]. Boruta has a strong ability to identify important genes from complex, high-dimensional genomic data. Its robust approach helps uncover significant genes that might be overlooked by other methods, such as LASSO, Elastic net, or feature selection techniques based on statistical tests [50,53]. The following steps were applied to identify the important genes:

Step 1: Shadow genes are generated by shuffling the value of the initial gene randomly.
Step 2: Merge the original genes and the shadow genes into a single dataset.
Step 3: Train RF-based classifier on the merged dataset and mean decrease accuracy was used to evaluate the importance of each gene.
Step 4: Calculate the Z-score for each gene by utilizing the gene’s importance values.
Step 5: Genes exceeding a specific threshold Z-score (typically positive) are labeled as "Confirmed," while genes falling below this threshold are labeled as "Rejected."
Step 6: Continue this process until all genes are either confirmed or rejected.

The ’Boruta’ package in R programming was utilized to identify the important genes of NSCLC.

### Determination of core genes

The core genes were determined by overlapping the genes obtained from the PPI network, module analysis, and ML-based approach. The computational formula is as follows:


$$\begin{array}{c} Core\;Genes=\overset{r}{\underset{i=1}{⋂}}{Optimal\;genes\;identi\mathrm{fi}cation\;methods}_{i} \end{array}$$ (2)

here, r=3.

### Validation of core genes

This study validated the performance of core genes by two viewpoints: survival analysis and discriminative power analysis which are briefly described in the following subsections.

#### Survival analysis.

The survival analysis was performed to assess the prognostic significance of core genes using GEPIA (http://gepia.cancer-pku.cn/) [54,55]. GEPIA is an online-based bioinformatics tool that extracted data from the TCGA [56]. We divided the patients into low and high-risk groups based on their median gene expression (MGE) value. A patient was classified as a high-risk group if their gene expression value exceeded the MGE values and vice-versa. The significant difference in genes between the two groups was examined by hazard-ratio (HR) and log-rank test. We considered the core genes as prognostic genes using p-value  < 0.05.

#### Discriminative power analysis.

The discriminative power of the prognostic genes was evaluated using the test set. We trained a 1-dimensional convolutional neural network (1DCNN) for each prognostic gene and computed their area under the curve (AUC) value from the ROC curve [57,58]. Keras and Scikit-learn in Python were utilized for ROC analysis. We considered the prognostic genes as potential candidate biomarkers that yield an AUC value of more than 0.90 [59,60].

### Regulatory network analysis

The regulatory analysis of transcription factors (TFs) and microRNAs (miRNAs) for the potential biomarkers was performed to investigate the key candidate TFs and miRNAs that regulate gene expression at both the transcriptional and post-transcriptional levels. We conducted regularity network analysis of the potential biomarkers using network analyst-based web tool (https://www.networkanalyst.ca/) [61]. The key candidates TFs and miRNAs were selected through Cytoscape by employing Deg [62] and Betn [63].

### Drug gene interaction analysis

The drug genes interaction analysis was executed to explore the candidate drugs of the potential biomarkers for the treatment of NSCLC patients. This analysis was carried out using Drug-Gene Interaction database (DGIdb) (https://www.dgidb.org/) [64]. DGIdb is a comprehensive resource that provides information on the interactions between drugs and druggable genes [65].

## Experimental results

### Identification of DEGs

**Fig 2:**
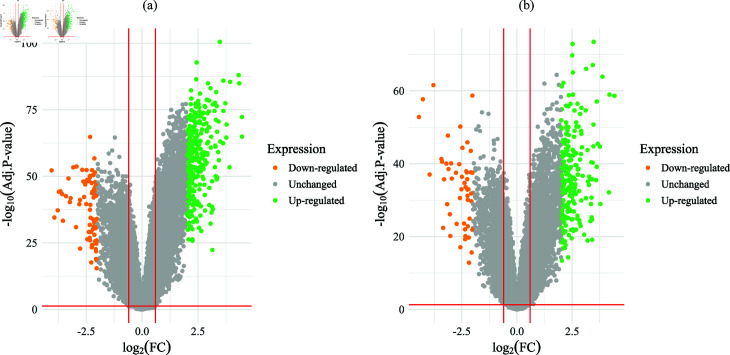
Volcano plot of the DEGs: (a) USA Cohort and (b) Taiwan cohort.

The DEGs were identified from the combined dataset based on the Adj. p-value  < 0.01 and $|log_{2}$ FC | > 2. As per the criteria, we identified 394 (318 up-regulated and 76 down-regulated) DEGs for USA cohort. The volcano plot of the DEGs between the NSCLC patients and healthy control for USA cohort is displayed in Fig 2a. Similarly, we also obtained a total of 277 (226 up-regulated and 51 down-regulated) DEGs for Taiwan cohort as shown in in Fig 2b.

### Carcinoma associated genes in NSCLC

The disease gene association analysis revealed that 64 genes out of 394 were associated with carcinoma in the NSCLC for USA cohort. The carcinoma associated genes are as follows: *TOP2A, ROBO4, MT1M, TCF21, HMGB3, AQP4, CXCL13, FGF2, THBD, EDNRB, DACH1, PEBP4, NUF2, PCLAF, LEPR, NEK2, ADAMTS8, SOX7, SEMA6A, MME, MMP1, SFTPC, TNNC1, SFTPD, CACNA2D2, MMP12, TGFBR3, CEACAM5, ADAM12, CLDN18, BIRC5, AFAP1-AS1, HPGD, EPAS1, PLA2G1B, UHRF1, COL11A1, LPL, HMMR, AGER, WIF1, KISS1R, SPP1, CTHRC1, GDF10, NTRK2, RRM2, ANGPT1, NEBL, ZBTB16, CAV1, SMAD6, KLF4, ANLN, TPX2, GJB2, RGCC, CYP24A1, PSAT1, SCGB1A1, FOSB, SFTPA1, TEK, and FGFR4*. Similarly, we also obtained 44 carcinoma associated genes for Taiwan cohort are as follows: *SFTPA2, MT1M, PINX1, SIX1, TCF21, AQP4, CXCL13, THBS2, CXCL14, AGER, THBD, CST1, EDNRB, DACH1, ADAMTS1, WIF1, PEBP4, PCLAF, SPP1, SOX7, ABCC3, MME, MMP1, ROBO4, ZBTB16, TNNC1, SFTPC, SFTPD, VEGFD, KLF4, FRMD3, TGFBR3, MMP12, ANLN, MMP11, GJB2, IL6, RGCC, PSAT1, CEACAM5, SCGB1A1, TEK, SFTPA1, AFAP1-AS1.* These carcinomas associated genes were utilized for the construction of PPI network, hub gene selection, module analysis, and important genes identification in ML-based approach, which are more clearly explained in the following subsections.

### Enrichment analysis of the carcinoma associated genes

Enrichment analysis was performed on the carcinoma associated genes in NSCLC for USA cohort and selected significantly associated top five GO terms and KEGG pathway. The BP result showed that the genes were significantly enriched in extracellular matrix organization, extracellular structure organization, regulation of DNA biosynthetic process, tissue remodeling, respiratory gaseous exchange by respiratory system. In CC, collagen trimer, clathrin-coated endocytic vesicle, multivesicular body, basolateral plasma membrane, lamellar body, and in MF, glycosaminoglycan binding, heparin binding, sulfur compound binding, metalloendopeptidase activity, fibroblast growth factor binding. The results of BP, CC, and MF are presented in Table 2. The KEGG pathway results revealed that the genes were enriched in Ras signaling pathway, MAPK signaling pathway, Calcium signaling pathway, PI3K-Akt signaling pathway and Rheumatoid arthritis as shown in Table 3.

**Table 2:** GO analysis for carcinoma associated DEGs for USA cohort. Top 5 items were selected.

Category	ID	Description	Count	p-value
BP	GO:0030198	Extracellular matrix organization	10	5.90E-07
	GO:0043062	Extracellular structure organization	10	6.00E-07
	GO:2000278	Regulation of DNA biosynthetic process	6	1.50E-06
	GO:0048771	Tissue remodeling	7	1.90E-06
	GO:0007585	Respiratory gaseous exchange by respiratory system	5	2.50E-06
CC	GO:0005581	Collagen trimer	4	1.80E-04
	GO:0045334	Clathrin-coated endocytic vesicle	3	9.10E-04
	GO:0005771	Multivesicular body	3	1.05E-03
	GO:0016323	Basolateral plasma membrane	5	1.17E-03
	GO:0042599	Lamellar body	2	1.35E-03
MF	GO:0005539	Glycosaminoglycan binding	8	1.20E-06
	GO:0008201	Heparin binding	7	1.80E-06
	GO:1901681	Sulfur compound binding	7	3.10E-05
	GO:0004222	Metalloendopeptidase activity	5	3.40E-05
	GO:0030198	Extracellular matrix organization	10	5.90E-07

**Table 3:** KEGG pathway analysis for carcinoma associated DEGs for USA cohort. Top 5 items were selected.

ID	Description	Count	p-value
hsa04014	Ras signaling pathway	6	8.29E-04
hsa04010	MAPK signaling pathway	6	2.71E-03
hsa04020	Calcium signaling pathway	5	6.70E-03
hsa04151	PI3K-Akt signaling pathway	6	6.79E-03
hsa05323	Rheumatoid arthritis	3	9.89E-03

### PPI network construction and hub genes selection

**Fig 3:**
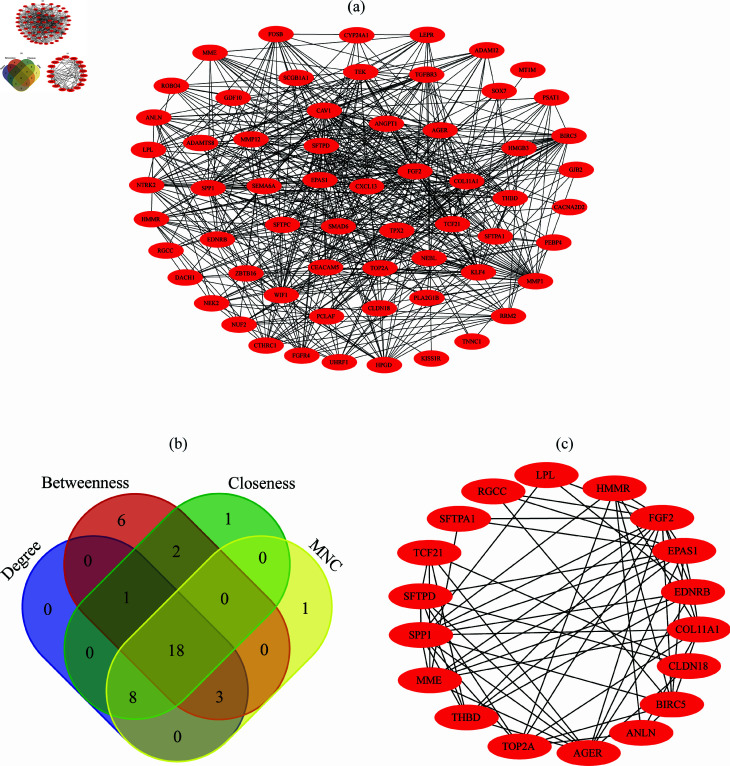
Analysis of PPI network and identification of core hub genes for USA cohort: (a) PPI network analysis for the carcinoma associated genes; (b) Identification of hub genes among four measures (Degree, Betweenness, Closeness, and MNC); and (c) PPI network analysis of identifying core hub genes.

We constructed a PPI network using 64 carcinoma associated DEGs for USA cohort as shown in Fig 3a. To identify the core hub genes, we used multiple network centrality measures, namely Deg, Betn, Clos, and MNC. These measures help identify genes that play pivotal roles in the network, either through direct interactions, connecting multiple pathways, or influencing other genes within the network. The PPI network consisted of 62 nodes (genes) and 524 edges (interactions), with an average Deg of 5.43. We selected top 30 DEGs based on each measure and found 18 overlapping genes across all four measures as shown in Fig 3b. These 18 genes were identified as core hub genes for USA cohort. The list of overlapping hub genes includes: *SFTPA1, AGER, TOP2A, SFTPD, COL11A1, CLDN18, EPAS1, SPP1, MME, HMMR, FGF2, EDNRB, TCF21, RGCC, THBD, LPL, ANLN, and BIRC* as core hub genes. The rank and degree of connectivity for each identified core hub genes for USA cohort is presented in Table 4. Similarly, we also constructed PPI network on 44 carcinoma associated DEGs for Taiwan cohort as shown in Fig 3c. We chose top 30 of each topological measure and found 16 overlapping genes, including *SIX1, ROBO4, CEACAM5, TEK, SPP1, AGER, TCF21, EDNRB, SFTPA2, ANLN, SCGB1A1, MMP1, MME, ABCC3, IL6, THBS2*.

**Table 4:** Degree of connectivity for the selected core hub genes in the USA cohort.

Hub Genes	Degree of Connectivity
FGF2	13
SPP1	12
EPAS1	10
AGER	9
BIRC5	8
COL11A1	8
EDNRB	8
MME	8
TOP2A	7
THBD	7
SFTPD	7
HMMR	6
TCF21	5
ANLN	4
SFTPA1	3
LPL	3
RGCC	3
CLDN18	3

### Module identification and its associated genes

**Fig 4:**
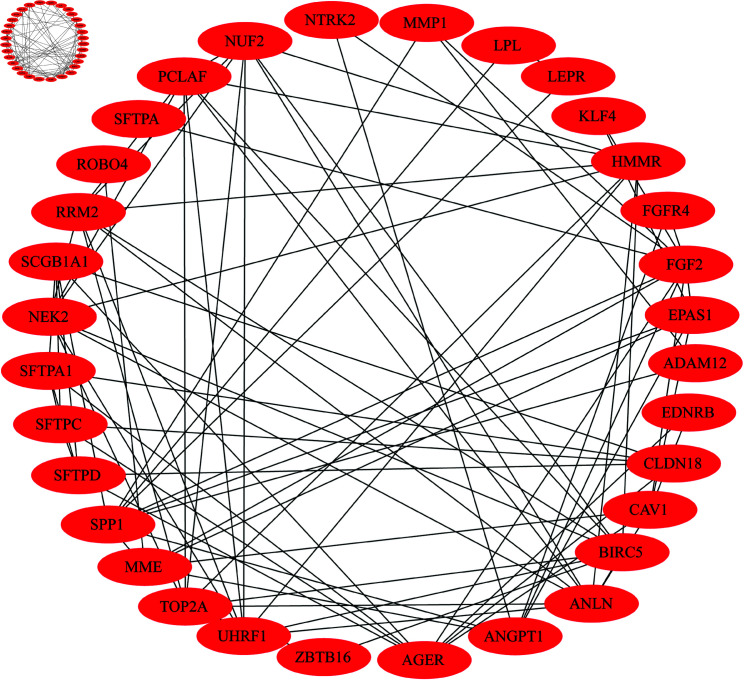
PPI network analysis of the hub genes for USA cohort.

We used MCODE for module or cluster analysis and got three clusters or modules based on the MCODE scores ranging from 3 to 6. We selected 2 modules for USA cohort as its MCODE scores value of more than 5. The 1st module contains 32(*FGFR4, SCGB1A1, SFTPA1, LPL, MMP1, NTRK2, ADAM12, FGF2, NUF2, AGER, LEPR, HMMR, EPAS1, ROBO4, ANGPT1, TOP2A, KLF4, SPP1, SFTPA1, NEK2, RRM2, EDNRB, BIRC5, SFTPD, UHRF1, PCLAF, ANLN, CLDN18, ZBTB16, CAV1, TEK and SFTPA*) genes, which had 32 nodes and 98 edges. Whereas, the 2nd module contains 10 (*ANGPT1, SCGB1A1, CLDN18, SPP1, SFTPA1, MME, SFTPD, AGER, SFTPC, EPAS1*) genes, which had 10 nodes and 27 edges. Following the union of two modules, we identified a total of 32 hub genes are as follows: (*FGFR4, RRM2, ANGPT1, AGER, NUF2, CLDN18, LEPR, PCLAF, EDNRB, TOP2A, SCGB1A1, NTRK2, ROBO4, SFTPA1, CAV1, ADAM12, EPAS1, LPL, SPP1, MME, SFTPD, HMMR, SFTPC, FGF2, MMP1, ZBTB16, KLF4, SFTPA, NEK2, ANLN, BIRC5, *and* UHRF1* and their PPI networks are illustrated in Fig 4. Similarly, we selected module 1 and module 2 for the Taiwan cohort. Following that, we found a total of 20 genes by combining the genes obtained from module 1 and module 2. These 20 genes are as foloows: *SFTPC, SFTPA2, SFTPA1, SIX1, SFTPD, AGER, SFTPA1, CAV1, MMP1, SFTPC, RGCC, MMP12, SPP1, MME, AGER, CXCL13, EDNRB, SFTPA2, MMP11,*and* THBS2*.

### Important genes identification using ML-based approach

The Boruta based machine learning approach identified 56 DEGs for NSCLC. The 56 DEGs are called ML-based important genes include *TOP2A, ROBO4, MT1M, TCF21, HMGB3, AQP4, CXCL13, THBD, EDNRB, DACH1, PEBP4, NUF2, PCLAF, LEPR, ADAMTS8, SOX7, SEMA6A, MME, MMP1, SFTPC, TNNC1, SFTPD, CACNA2D2, MMP12, TGFBR3, ADAM12, CLDN18, BIRC5, AFAP1-AS1, HPGD, UHRF1, COL11A1, LPL, HMMR, AGER, WIF1, KISS1R, SPP1, CTHRC1, GDF10, NTRK2, RRM2, SFTPA1, NEBL, CAV1, SMAD6, KLF4, ANLN, GJB2, RGCC, CYP24A1, PSAT1, SCGB1A1, SFTPA1, TEK, FGFR4*. Similarly, we identifed 41 DEGs for Taiwan cohort, including *MT1M, PINX1, SIX1, TCF21, AQP4, CXCL13, THBS2, AGER, THBD, CST1, EDNRB, DACH1, ADAMTS1, WIF1, PEBP4, PCLAF, SPP1, SOX7, ABCC3, MME, MMP1, ROBO4, ZBTB16, TNNC1, SFTPC, SFTPD, VEGFD, KLF4, FRMD3, TGFBR3, MMP12, ANLN, MMP11, GJB2, IL6, RGCC, PSAT1, CEACAM5, SCGB1A1, TEK, *and* AFAP1-AS1*.

### Identification of core genes

**Fig 5:**
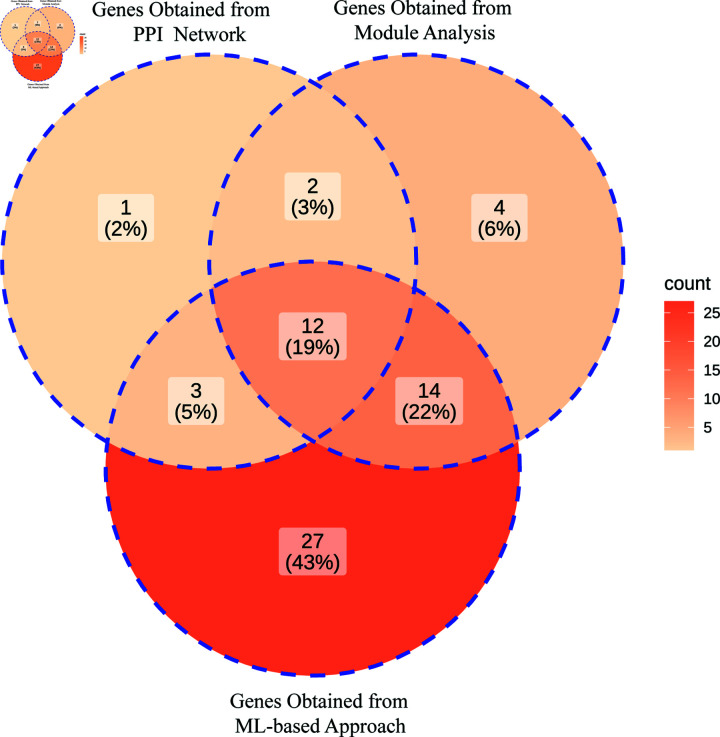
Identification of core genes for USA cohort by intersecting genes, obtained from PPI network, Module analysis, and ML-based approach. The depth of the color corresponds to the quantity of hub genes present.

We identified 12 common genes for USA cohort by intersecting 18 genes, obtained from PPI network, 32 genes obtained from module analysis, and 56 genes obtained from ML-based approach, as illustrated in Fig 5. The identified 12 genes (*CLDN18, AGER, EDNRB, TOP2A, MME, SPP1, LPL, SFTPD, HMMR, SFTPA1, ANLN, and BIRC5*) were considered as core genes for USA cohort. Similarly, we identified 10 core genes (*AGER, SIX1, EDNRB, MME, ABCC3, ROBO4, SPP1, TCF21, MMP1,* and* THBS2*) for Taiwan cohort.

### Identification of prognostic biomarkers

**Fig 6:**
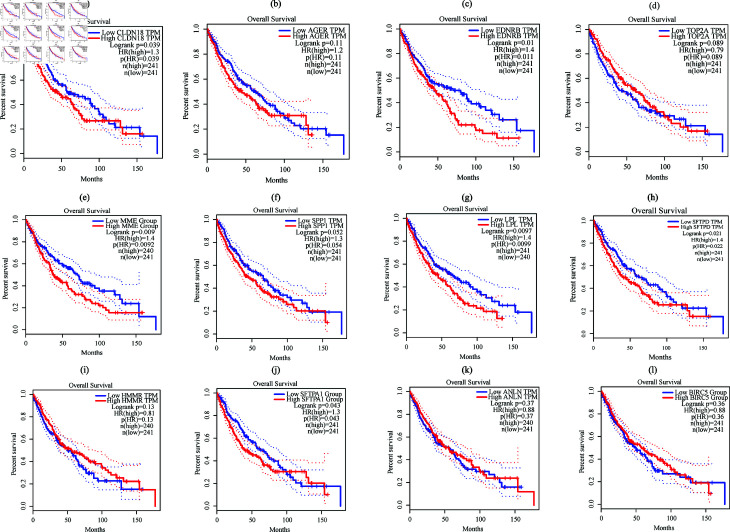
Survival analysis of 12 core genes for USA cohort: (a) *CLDN18*; (b) *AGER*; (c) *EDNRB*; (d) *TOP2A*; (e) *MME*; (f) *SPP1*; (g) *LPL*; (h) *SFTPD*; (i) *HMMR*; (j) *SFTPA1*; (k) *ANLN*; (l) *BIRC5.* The x-axis represents time to event (in days) and the y-axis represents survival probability.

The prognostic significance of 12 core genes for USA cohort was assessed by survival analysis based on the p-value  < 0.05 as shown in Fig 6. The results revealed that six core genes (*SFTPD, SFTPA1, LPL, CLDN18, EDNRB, *and* MME*) were significantly associated with the survival status of NSCLC patients (p < 0.05). These six genes (*SFTPD, SFTPA1, LPL, CLDN18, EDNRB, and ROBO4*) are considered as prognostic biomarkers for USA cohort. In the same way, we found 5 prognostic genes including *SIX1, EDNRB, MME, ROBO4,* and* TCF21* for Taiwan cohort.

**Fig 7:**
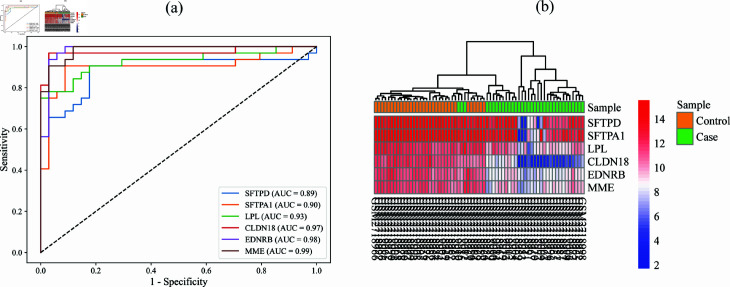
ROC curves and heatmap of six genes on test set for USA cohort (a) ROC curves and (b) Heatmap.

The discriminative power of the prognostic genes was evaluated by the AUC value based on the test set. Fig 7 displayed the ROC curves of six prognostic biomarkers for USA cohort and their corresponding heatmap. The AUC values of *LPL, CLDN18, EDNRB, MME* genes were as: 0.927 (95% CI: 0.857–0.976), 0.973 (95% CI: 0.929–1.00), 0.984 (95% CI: 0.962–1.00), 0.986 (95% CI: 0.967–0.999), respectively, while *SFTPD* and *SFTPA1* were 0.887 (95% CI: 0.804–0.957) and 0.897(95% CI:0.831-0.973). The findings indicated that the four biomarkers have more discriminative power for classifying cancer patients from healthy control (AUC > 0.90). This study declared these four biomarkers (*LPL, CLDN18, EDNRB, MME*)) as potential biomarkers for USA cohort. Similarly, we also computed the AUC values of each prognostic gene for Taiwan cohort and the AUC values of *EDNRB, MME, ROBO4* genes were as: 0.960 (95% CI: 0.857–1.00), 0.918 (95% CI: 0.846–0.986), 0.962 (95% CI: 0.876–1.00), respectively, while *SIX1* and *TCF21* were 0.854 (95% CI: 0.727–0.959) and 0.866 (95% CI: 0.739–0.963). The findings indicated that the three biomarkers have more discriminative power for classifying cancer patients from healthy control (AUC > 0.90). This study declared these three biomarkers (*EDNRB, MME, ROBO4*) as potential biomarkers for Taiwan cohort.

## Comparison of potential biomarkers between USA and Taiwan cohorts

**Fig 8:**
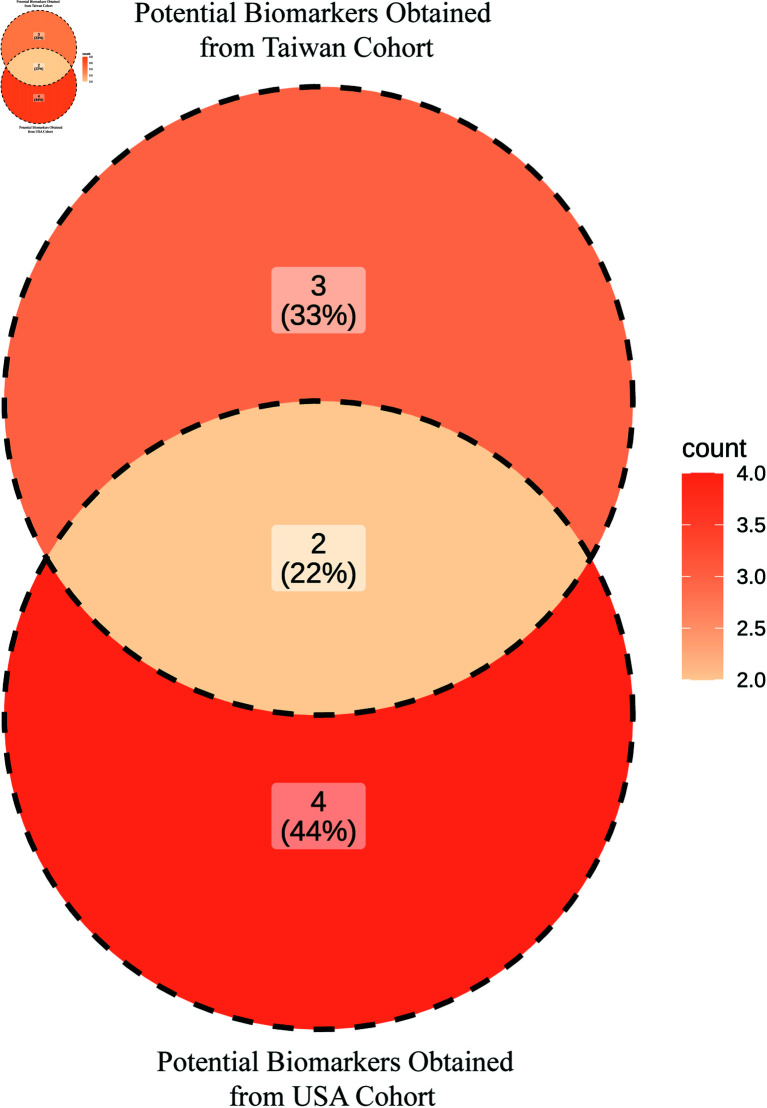
Identification of most promising potential biomarkers for NSCLC by intersecting genes, obtained from Taiwan cohort and USA cohort.

We identified four genes *(LPL, CLDN18, EDNRB, and MME)* as potential biomarkers for NSCLC in the USA cohort, and three genes *EDNRB, MME, and ROBO4* as potential biomarkers for NSCLC in the Taiwan cohort. To compare these potential biomarkers between USA and Taiwan cohorts, we performed an intersection analysis between the USA and Taiwan cohort genes. Finally, two genes *(EDNRB and MME)* were identified as common biomarkers across both cohorts, as shown in Fig 8. Therefore, this study designated *EDNRB and MME* as the most promising potential biomarkers for NSCLC.

### Regulatory analysis of potential biomarkers

**Fig 9:**
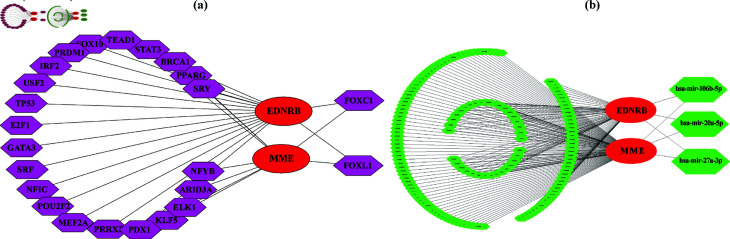
Regulatory network between potential biomarkers with TFs and miRNAs. The red, magenta and green color nodes represent the potential biomarkers, TFs, and miRNAs, respectively. (a) Potential biomarkers and TF interaction network, (b) Potential biomarkers and miRNA interaction network.

We constructed an interaction network between potential biomarkers vs. TFs to explore the candidate regulatory TFs for NSCLC patients. In this network, the potential biomarkers were represented by elliptical nodes, while the TFs were represented by circular nodes, as illustrated in Fig. 9a. The TFs-based regulatory analysis revealed that FOXC1 and FOXL1 are the top candidate TFs for the potential biomarkers that regulate the expression of NSCLC at the transcription level. Similarly, a separate interaction network between potential biomarkers vs. miRNAs was also constructed to examine the candidate miRNAs for NSCLC. The elliptical nodes in this network represent potential biomarkers, whereas the circular nodes represent the miRNAs, as shown in Fig 9b. The miRNAs-based regulatory analysis showed that hsa-mir-106b-5p, hsa-mir-20a-5p, and hsa-mir-27a-3p are the top candidate miRNAs that regulate the expression of NSCLC patients at the post-transcriptional level.

### Potential biomarkers with their associated drugs

**Fig 10:**
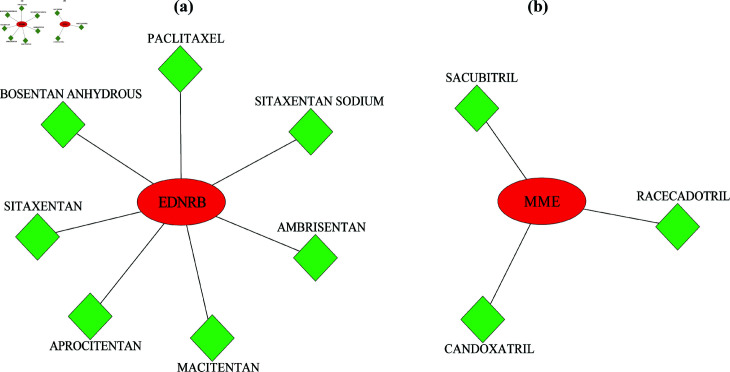
Potential biomarkers and drugs interaction netweork. The red and green color nodes represent the potential biomarkers and drugs. (a) EDNRB and Drugs interaction and (b) MME and Drugs interaction.

Using drug-gene interaction analysis, we identified key candidate drugs for the potential biomarkers, as illustrated in Fig 10. The green diamond represents the candidate drugs, while the red circle indicates the potential biomarkers. Our analysis revealed that a total of 7 (AMBRISENTAN, MACITENTAN, SITAXENTAN SODIUM, APROCITENTAN, PACLITAXEL, SITAXENTAN, BOSENTAN ANHYDROUS) drugs interact with *EDNRB*, and 3 (CANDOXATRIL, RACECADOTRIL, SACUBITRIL) drugs interact with *MME* of NSCLC, as illustrated in Fig 10. Based on the findings, we proposed that EDNRB and MME play central roles in the development of novel treatment targets for NSCLC, offering promising avenues for therapeutic intervention.

## Discussion

This study attempted to propose a system to identify potential biomarkers for patients with NSCLC using the integration of bioinformatics and ML-based approaches. In high-dimensional genomic data analysis, biomarker selection is challenging, mainly due to the large number of characteristics relative to the limited sample size. To identify effective biomarkers in these settings, multiple approaches are available, including hypothesis-based tests, penalized methods like the least absolute shrinkage and selection operator (LASSO), and other ML-based approaches such as support vector machine recursive feature elimination (SVMRFE). Hypotheses-based tests independently evaluate each biomarker, which means that they often ignore potential correlations between biomarkers, limiting their ability to capture complex biological interactions. Several studies have previously used ML-based approaches such as LASSO and/or SVMRFE to identify DEGs in NSCLC [9,66,67]. The LASSO method selects features by shrinking regression coefficients to zero, but it struggles with correlated features. When the irrepresentable condition (IC)—a covariance-related requirement—is violated, LASSO may fail to identify all relevant biomarkers, often selecting only one feature from correlated ones. on the other hand, SVMRFE tends to choose only one feature from groups of correlated genes, potentially missing important DEGs. Additionally, the performance of SVMRFE depends On model parameters, making it less stable for genomic datasets. In contrast, the Boruta method is more effective for data with complex feature interactions. It assesses feature importance without relying on the IC, making it robust against correlations and capable of identifying all relevant biomarkers, even when features are interdependent.

In this investigation, we used combined training dataset to identify the DEGs for NSCLC patients. We identified 394 DEGs for NSCLC and selected 64 carcinoma-associated genes from these 394 using DisGeNET. Enrichment analysis was performed on carcinoma-associated 64 DEGs and examined significant top 5 GO terms and KEGG pathways to better understand the mechanisms and progression of NSCLC patients. We observed that extracellular matrix organization is the most candidate BP that are strongly linked to the development and progression of NSCLC. This findings was coincided with the earlier sittings [68–70]. Extracellular matrix organization facilitates tumor invasion, metastasis, and angiogenesis, alters cell signaling to promote cancer growth, and creates a tumor microenvironment that aids in immune evasion. The CC-based GO term enriched in collagen trimer, which was corroborated with the prior studies [71–75]. The collagen trimer is essential for tissue strength and elasticity. The cell surface receptors in the lung’s mediate responses to environmental stimuli and pathogens [76,77]. The clathrin-coated endocytic vesicle is crucial for lung cell homeostasis and response to pathogens. In the case of MFs, glycosaminoglycan binding GO term influences lung cancer tissue remodeling and inflammatory responses. A recent study revealed that identical glycosaminoglycan binding is essential for understanding lung cancer mechanisms and developing treatments [78]. We also identified top 5 KEGG pathways that are closely related to NSCLC as shown in Table 3. The ras signaling pathway plays a crucial role in inflammatory lung cancer and holds potential as a therapeutic target [79,80]. The viral protein interaction with cytokine and cytokine receptor can lead to severe inflammatory reactions in the lungs. However, we constructed a PPI network on the carcinoma associated 64 genes using cytoscape. Within this network, the hub genes identified were *SFTPA1, AGER, TOP2A, SFTPD, COL11A1, CLDN18, EPAS1, SPP1, MME, HMMR, FGF2, EDNRB, TCF21, RGCC, THBD, LPL, ANLN, BIRC5*, as illustrated in Fig 5. The potential modules were determined based on the MCODE scores, ultimately selecting two modules. Thses modules include *FGFR4, RRM2, ANGPT1, AGER, NUF2, CLDN18, LEPR, PCLAF, EDNRB, TOP2A, SCGB1A1, NTRK2, ROBO4, SFTPA1, CAV1, ADAM12, EPAS1, LPL, SPP1, MME, SFTPD, HMMR, SFTPC, FGF2, MMP1, ZBTB16, KLF4, SFTPA, NEK2, ANLN, BIRC5, UHRF1* genes, within the PPI network as shown in Fig 6.

Additionally, using the Boruta technique, we identified *TOP2A, ROBO4, MT1M, TCF21, HMGB3, AQP4, CXCL13, THBD, EDNRB, DACH1, PEBP4, NUF2, PCLAF, LEPR, ADAMTS8, SOX7, SEMA6A, MME, MMP1, SFTPC, TNNC1, SFTPD, CACNA2D2, MMP12, TGFBR3, ADAM12, CLDN18, BIRC5, AFAP1-AS1, HPGD, UHRF1, COL11A1, LPL, HMMR, AGER, WIF1, KISS1R, SPP1, CTHRC1, GDF10, NTRK2, RRM2, SFTPA1, NEBL, CAV1, SMAD6, KLF4, ANLN, GJB2, RGCC, CYP24A1, PSAT1, SCGB1A1, SFTPA1, TEK, FGFR4*ML-based hub genes. We determined the core hub genes (*CLDN18, AGER, EDNRB, TOP2A, MME, SPP1, LPL, SFTPD, HMMR, SFTPA1, ANLN, BIRC5*) through the intersection of identified genes, obtained from PPI network analysis, module analysis, and ML-based approach, as illustrated in Fig 7. These genes are crucial in NSCLC pathology and may provide insights into novel therapeutic strategies and precision medicine approaches. We validated the core genes by two different ways, firstly, survival analysis was conducted to identify the prognostic biomarkers among the core genes and identified (*SFTPD, SFTPA1, LPL, CLDN18, EDNRB, MME*) as prognostic biomarkers (See in Fig 9). Secondly, the AUC value on the testing set was used to determine the discriminative power of the prognostic genes. It was observed that out of the six prognostic genes, four (*LPL, CLDN18, EDNRB, MME* ) achieved high discriminative power in classifying NSCLC patients from healthy controls (Fig 9). These findings demonstrated that four genes may serve as potential diagnostic biomarkers for NSCLC in USA cohorts. To identify common and region-specific biomarkers between the USA and Taiwan cohorts, we utilized three datasets from Taiwan, following the same protocol. This analysis revealed *EDNRB*, *MME*, and *ROBO4* as potential biomarkers for NSCLC in the Taiwan cohort. Finally, two biomarkers *(*EDNRB and MME*)* were found as common diagnostic potential biomarkers by intersecting genes, obtained from USA and Taiwan cohorts. Therefore, this study proposed *EDNRB* and *MME* as the most promising potential biomarkers for NSCLC.

Endothelin Receptor Type B (*EDNRB*) has been identified as a potential biomarker for NSCLC development and progression, aligning with findings from previous studies [81–84]. It is an essential gene that encodes a G protein-coupled receptor involved in regulating vasoconstriction, cell proliferation, and differentiation. It plays a crucial role in various physiological processes, including neural crest cell migration during embryonic development, melanocyte development, and the regulation of vascular tone in the cardiovascular system. The *EDNRB* biomarkers are additionally linked to breast cancer, colorectal cancer, and prostate cancer [85–87]. The targeting of EDNRB and its associated signaling pathways could be serve as a potential therapeutic strategy for effectively managing lung cancer [88].

Membrane Metalloendopeptidase (*MME*), also known as *CD10*, is a cell surface protein that cleaves and inactivates peptide hormones involved in various physiological processes. *CD10* expression in NSCLC is observed in both epithelial and stromal cells, playing distinct roles in tumor biology [89,90]. In epithelial cells, *CD10* expression is often associated with tumor cell aggressiveness, promoting tumor proliferation, invasion, and metastasis. Its presence in epithelial tumor cells can indicate a more invasive phenotype, contributing to cancer progression. In stromal cells, *CD10* expression influences the tumor microenvironment by modulating interactions between cancer cells and the surrounding stromal tissue. This can affect processes such as angiogenesis, immune response, and extracellular matrix remodeling, ultimately supporting tumor growth and metastasis. A high *CD10*+/low *CD20*+ immune cell infiltration ratio has been identified as a significant prognostic factor for lung carcinoma [91]. This suggests that an elevated presence of *CD10*+ cells is associated with poorer patient outcomes. Several studies shown that *MME* downregulation is strongly associated with several cancer types, including breast [92], colon [93], bladder urothelial carcinoma [94], and colorectal [93]. This decrease in *MME* expression may disrupt cell-cell and cell-matrix interactions, facilitating tumor cell migration and invasion key processes in cancer metastasis. This study identified key TFs, FOXC1 and FOXL1, as well as miRNAs, hsa-mir-106b-5p, hsa-mir-20a-5p, and hsa-mir-27a-3p. These regulators, influenced by EDNRB and MME, play essential roles in the development and progression of NSCLC. FOXC1 is a transcription factor involved in the development of lymphatic vessels, arterial cell specification, and cardiovascular development [95,96]. *EDNRB* contributes to the development of vascular and lymphatic systems by influencing endothelial cell behavior, while *MME* plays a role in cardiovascular homeostasis by regulating vascular function. Together, these interactions highlight the critical role of FOXC1 in the proper formation and specification of the cardiovascular and lymphatic systems [97]. Moreover, FOXL1 is a TF that regulates several critical cellular functions essential for lung cell development and function. It plays a key role in differentiation, influencing the maturation and specialization of lung epithelial cells [98]. Furthermore, FOXL1 is implicated in regulating apoptosis, the process of programmed cell death, which is crucial for eliminating damaged or excess cells in the lung tissue. Dysregulation of FOXL1 can contribute to pathological conditions, including lung cancer, highlighting its importance in lung biology. The expression of hsa-miR-106b-5p in the serum of NSCLC patients has significant clinical implications, as elevated levels are associated with poor prognosis, suggesting its potential as a biomarker for disease progression and outcome. Studies indicated that miR-106b-5p may promote tumor growth and metastasis by targeting various tumor suppressor genes and signaling pathways [99,100]. Its expression levels can reflect tumor burden and correlate with clinical parameters such as tumor size, lymph node involvement, and overall survival rates. Therefore, miR-106b-5p holds promise as a non-invasive biomarker for diagnosing and monitoring NSCLC, aiding in the assessment of treatment response and guiding therapeutic decisions. The hsa-muir-20a-5p regulator suppresses tumor angiogenesis in NSCLC by targeting the RRM2-mediated PI3K/Akt signaling pathway [101]. By inhibiting RRM2, microRNA-20a-5p disrupts the activation of the PI3K/Akt pathway, which is crucial for promoting angiogenesis and tumor growth. It has prognostic significance also SCLC and prostate cancer [102,103]. The hsa-mir-27a-3p plays critical roles in lung cancer progression by promoting tumor growth, enhancing metastatic potential, stimulating angiogenesis, inhibiting apoptosis, and contributing to treatment resistance [104]. Additionally, it is important in invasion, metastasis, and epithelial-mesenchymal transition in hepatocellular carcinoma, highlighting its broader implications in cancer biology. The findings highlight the complex regulatory network involving these TFs and miRNAs, shedding light on their potential roles in NSCLC pathology. This study suggested ten drugs that interact with *EDNRB* and *MME*, presenting a promising avenue for developing new therapeutic targets for NSCLC. These interactions suggest that targeting *EDNRB* and *MME* might be beneficial for therapeutic strategies, enhancing the understanding of NSCLC’s and play a crucial role in advancing treatment strategies and improving the effectiveness of targeted therapies for patients with NSCLC.

## Conclusion

This study aimed to identify the potential biomarkers for lung cancer using integrated bioinformatics and ML-based approaches. After performing different bioinformatics and ML-based analyses, our findings indicated that *EDNRB* and *MME* are the potential biomarkers for NSCLC between USA and Taiwan cohorts. The potential biomarkers regulatory network analysis revealed that the key TFs (FOXC1 and FOXL1) and miRNAs (hsa-mir-106b-5p, hsa-mir-20a-5p, and hsa-mir-27a-3p) as the transcriptional and post-transcriptional regulators of NSCLC. Additionally, this study explored candidate drugs for potential NSCLC biomarkers, highlighting therapeutic agents that interact with them and offering insights into treatment options. Therefore, the findings of this study offer substantial potential to improve NSCLC diagnosis by identifying reliable biomarkers and guiding the development of targeted therapies. These advancements can help physicians design more effective treatment plans for NSCLC and may reduce healthcare costs by enabling early detection and preventing disease progression.
